# Perspektiven der Landesinstitute zur Steuerung digitalisierungsbezogener Professionalisierung von Schulleitungen zwischen Erwartung und Unterstützung: „Aber wenn er nicht ‚Piep‘ sagt, dann kommen wir nicht.“

**DOI:** 10.1007/s11612-022-00656-3

**Published:** 2022-11-28

**Authors:** Christoph Kruse, Ella Grigoleit, Pierre Tulowitzki

**Affiliations:** 1 Institut für Erziehungswissenschaft, Westfälische Wilhelms-Universität Münster, Bispinghof 5/6, 48143 Münster, Deutschland; 2 Institut Weiterbildung und Beratung, Pädagogische Hochschule FHNW, Bahnhofstrasse 6, 5210 Windisch, Schweiz

**Keywords:** Schulleitung, Schulentwicklung, Digitalisierung, Professionalisierung, Landesinstitute, School leadership, School development, Digitization, Professionalization, (Federal) state institutes

## Abstract

In diesem Artikel der Zeitschrift Gruppe. Interaktion. Organisation. werden auf Basis von Daten aus einer Interviewstudie die Perspektiven von Landesinstituten für Schulen (LiS) auf die Schnittstelle zur Schulleitung im Rahmen digitalisierungsbezogener Schulentwicklung analysiert. Die LiS verantworten in diesem Kontext bedeutsame Professionalisierungsmaßnahmen für Schulleitungen. In der Analyseperspektive der Educational Governance werden die LiS bzgl. der Schnittstelle in einen doppelten Fokus genommen: Zum einen stellt sich die Herausforderung einer gelingenden Passung von Qualifizierungsmaßnahmen an der Schnittstelle von LiS und Schulleitung (sowohl inhaltlich als auch organisatorisch), sodass schulische Führungskräfte in ihrer digitalisierungsbezogenen Kompetenzentwicklung effektiv unterstützt werden können. Zum anderen kann diese Schnittstelle mit Fokus auf die Steuerung und Implementation gelingender digitalisierungsbezogener Schulentwicklung innerhalb des Bildungssystems analysiert werden. Das Ziel der Studie liegt darin, Relevanzsetzungen (im Sinne von Handlungslogiken) der LiS in der Gestaltung der Professionalisierungsangebote zu identifizieren und auf die Herstellung von Passung an der Schnittstelle (im Sinne gelingender Handlungskoordination) zu diskutieren. Es zeigt sich, dass die Handlungslogik der LiS u. a. von Steuerungsimpulsen des übergeordneten Ministeriums, aber maßgeblich auch von normativen Erwartungen sowie Anpassungsstrategien an die (wahrgenommenen) Handlungslogiken der Schulleitungen beeinflusst ist. Für die Steuerung und Handlungskoordination kommt insofern nicht nur (unilateral) den LiS, sondern (wechselseitig) auch der Schulleitung eine zentrale Bedeutung für die Gestaltung und potenzielle Wirkung der digitalisierungsbezogenen Professionalisierungsangebote zu. Hieran anschließende Probleme, Herausforderungen und Implikationen für die Forschung und (Steuerung der) Praxis werden diskutiert.

## Abstract (en)

This paper in the journal Gruppe. Interaktion. Organisation. provides insights into the perspectives of state institutes for teacher training and professional development (LiS) regarding the interface to school leadership in the scope of digitization-related school development using interview data. In this context, the LiS take responsibility for relevant professionalization measures for school leadership. Following a governance paradigm, the function of the LiS regarding the interface comes into a twofold focus: On the one hand, the challenge of a successful fit of qualification measures at the interface of state institutes and school leadership (in terms of both content and organization) arises, so that school leaders can be effectively supported in their digitization-related competence development. On the other hand, this interface can be analyzed with a focus on the management and implementation of successful digitization-related school development within the education system. The aim of the study is to identify approaches to relevance (in the sense of operational logics) of the LiS in the design of professionalization measures and to discuss the creation of a fit at the interface level (in the sense of successful operational coordination). It becomes apparent that the logic of action of the LiS is influenced, among other things, by control impulses of the superordinate ministry, but also significantly by role expectations and adaptation strategies to the (perceived) logic of action of school leadership. For the control and coordination of action, not only (unilaterally) the LiS, but also (reciprocally) the school leadership is of significance for the design and potential effect of digitization-related professionalization opportunities. Subsequent problems, challenges and implications for research and (steering of) practice are discussed.

## Einleitung

Schulleitungen kommt innerhalb der Organisation Schule eine Schlüsselrolle zu – sowohl im schulischen Alltag als auch in Schulentwicklungsprozessen (Bonsen 2016). Die im Zuge der Pandemie verstärkte Digitalisierung von Schule fordert Schulleitungen als „Change Agents“ besonders heraus (Feldhoff et al. 2022; Tulowitzki und Gerick 2020) und sie spielen auch bei der Umsetzung eine zentrale Rolle (Prasse 2012). Die Digitalisierung wird dabei „als relevant für den gesamten Prozess der Schulentwicklung“ betrachtet, die „als Querschnitt alle Aufgaben von Schulleitungen“ (Schiefner-Rohs 2016, S. 1402) betrifft. Dabei geht es somit nicht nur um die Unterrichtsebene, sondern auch um eine umfassende organisationale Adaption an veränderte Rahmenbedingungen und Anforderungen an Schule, z. B. um die Digitalisierung von Management- und HR-Prozessen, Wissensmanagement und Kommunikation. Ferner gaben in den Pandemiejahren 2020–2021 viele Schulleitungen an, dass verschiedene Facetten der Digitalisierung (z. B. heterogene digitalisierungsbezogene Kompetenz im Kollegium, Entwicklung und Implementation von Konzepten für digitales Lernen) eine große Bedeutung in ihrem Arbeitsleben einnahmen und z. T. auch mit starken Belastungen verbunden waren (Feldhoff et al. 2022). Auch wurde ein großer Bedarf nach der Erweiterung digitalisierungsbezogener Kompetenzen zum Ausdruck gebracht (z. B. Pikowsky et al. 2020).

Diesbezügliche Professionalisierungsmaßnahmen für Schulleitende werden in Deutschland oftmals von den Landesinstituten für Schule (LiS; genaue Bezeichnung variiert je Bundesland), verantwortet und gesteuert. In der vorgestellten Studie wird die Interaktion an der Schnittstelle zwischen der Organisation LiS (repräsentiert durch die zuständigen Akteure für die digitalisierungsbezogene Schulleitungsqualifizierung) und der Organisation Schule (repräsentiert durch den Akteur der Schulleitung) aus der Perspektive der LiS in den Blick genommen. Die Interaktion wird in der Analyse als Handlungskoordination mit akteurspezifischer Handlungslogik gefasst. Indem die Akteure LiS und Schulleitung in der Perspektive der Educational Governance verortet und anschließend bezüglich aktueller Forschungsbefunde betrachtet werden, gerät die Schnittstelle in einen doppelten Fokus: Zum einen kann sie bezüglich der inhaltlichen und auch organisatorischen Passung von Qualifizierungsmaßnahmen und zum anderen mit Fokus auf die Steuerung und Implementation gelingender digitalisierungsbezogener Schulentwicklung analysiert werden. Vor dem Hintergrund dieser interorganisationalen Herausforderungen werden die Fragestellung und das Forschungsdesign abgeleitet, die Ergebnisse der Studie zur Handlungslogik der LiS berichtet und bezüglich des Theorie- und Forschungsstands diskutiert. Der Beitrag schließt mit einem Fazit mit Implikationen für die unterschiedlichen Akteure.

## Theoretischer Hintergrund

### Rahmenkonzept Educational Governance

Unter Bezugnahme auf die Governance-Forschung wird in dieser Studie das Phänomen der Schulentwicklung mit dem Begriff der Handlungskoordination analytisch in den Bick genommen. Mit diesem „nicht wertenden Begriff von ‚Koordination‘ werden […] die Art und Funktionalität des Zusammenwirkens der verschiedenen Akteure analysiert, ohne vorauszusetzen, wer ‚steuert‘ und wer höchstens als ‚Widerstandsfaktor‘ einzukalkulieren ist“ (Altrichter und Maag Merki 2016, S. 9). Gemäß dieser Analyseperspektive geschieht die Entwicklung von Einzelschulen in verschiedenen Akteurskonstellationen unter wechselseitiger Beeinflussung. Diese Perspektive birgt für die hier vorgestellte Studie die Chance, „das Handeln der Akteure sowie die gegenseitigen Abhängigkeiten im Mehrebenensystem differenzierter zu beschreiben und dabei bestimmte Koordinationsdefizite oder -leistungen sichtbar zu machen“ (Altrichter und Maag Merki 2016, S. 8). Dieses Mehrebenensystem strukturiert sich im schulischen Sektor durch eine Makro- (Gesetzgebung und -durchsetzung: Ministerium und Schulaufsicht), Meso- (Einzelschule: hier insbesondere Schulleitung) und Mikroebene (Unterricht: einzelne Lehrkraft). Bezugnehmend auf den systemtheoretisch fundierten Begriff der „Steuerungs-Illusion“ (Rolff 2002, S. 79) verweist Bonsen (2016) auf die Fragwürdigkeit zielgenauer Steuerung schulischer Prozesse (für diese Studie: digitalisierungsbezogene Schulentwicklungsprozesse) durch Akteure oder Institutionen der Makroebene des Systems. Somit lenkt diese Perspektive „die Aufmerksamkeit auf Fragen grenzüberschreitender Koordination zwischen ‚Systemebenen‘ […]: Die Analyse muss Prozesse und Effekte auf unterschiedlichen Ebenen berücksichtigen; die Intervention sieht sich mit einer Reihe von ‚Schnittstellenproblemen‘ konfrontiert, die sich aus den unterschiedlichen Handlungslogiken, Werthierarchien, ‚Sprachen‘ und Aufmerksamkeitsprioritäten der ‚Ebenen‘ ergeben“ (Altrichter und Maag Merki 2016, S. 11). Der Begriff der Handlungslogik umfasst hierbei „verschiedene Relevanzkriterien und Informationsanforderungen, innerhalb derer die Akteure auf unterschiedliche Weise Informationen und Wissen generieren, ausdeuten, gewichten und verteilen“ (Kussau und Brüsemeister 2007, S. 33).

**Figure 1:**
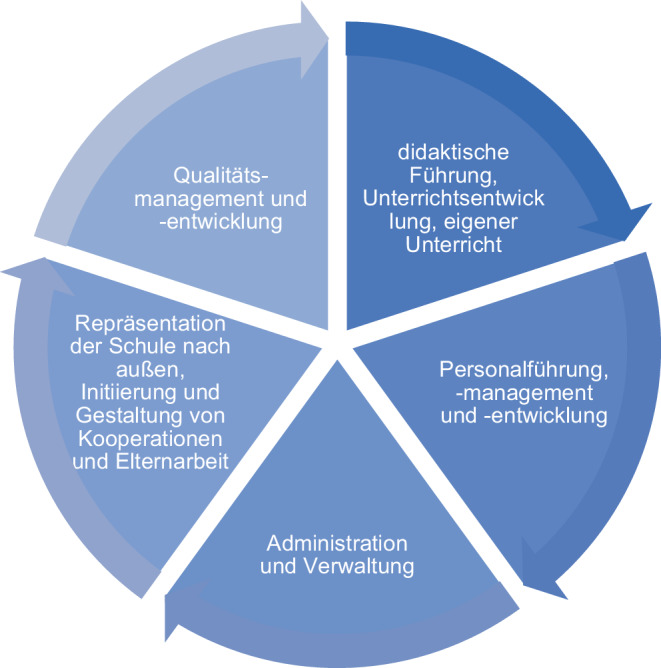

Mit der Notwendigkeit schulischer Adaptionsprozesse an die fortschreitende Digitalisierung geraten Schulleitungen mit ihrer akteurspezifischen Handlungslogik auf der Mesoebene in besonderer Weise in den Blick. Dass diese Logik nicht direkt von außen gesteuert werden kann und von individuellen wie überindividuellen Faktoren abhängig ist, verdeutlicht Bonsen (2016): Die „nicht immer spannungsfreie Vermittlungs- und Kontrollposition zwischen Administration und Kollegium“ (Bonsen 2016, S. 302) ist selbst in besonderer Weise gefordert, die organisationale Entwicklung der eigenen Schule zu steuern. Im Kontext der Digitalisierung besteht das Ziel dieser Steuerung nun darin, die Qualität schulischer Bildung digitalisierungsbezogen zu entwickeln (zu allgemeinen Aufgaben und Tätigkeiten von Schulleitungen siehe Kap. 2.2.2 und Abb. 1). Dem hierbei entstehenden Professionalisierungsbedarf der Schulleitung (vgl. Kap. 2.2.2) begegnen im staatlichen Bildungssystem vornehmlich die LiS. Diese sind grundsätzlich dem jeweiligen Landesministerium untergeordnet und insofern weisungsgebunden (Rürup 2014). Die Aufgabe der LiS, die in diesem Beitrag in den Blick rückt, ist die der digitalisierungsbezogenen Professionalisierung von Schulleitungen im Sinne einer Angebotsgestaltung und -durchführung^1^. Im Mehrebenensystem verortet Altrichter (2019) die LiS auf intermediärer Ebene mit einer Vermittlungs- und Koordinationsfunktion zwischen allen o. g. Ebenen, wobei die Fortbildung „aber weniger als Hilfe zu einer allgemeinen Höherqualifizierung […] sondern als intermediäres Unterstützungssystem für die angezielten Veränderungen der Systemsteuerung gefordert“ (Altrichter 2019, S. 72) werde. Das verdeutlicht die Governance-Perspektive auf die so als Akteur im Mehrebenensystem konzipierten LiS, die damit nicht nur mit ihrer Qualifizierungs- sondern auch mit ihrer Steuerungsfunktion in den forschungsanalytischen Blick geraten.

Aus den Aufgaben der beiden Akteure LiS und Schulleitung abgeleitet lässt sich nun das Zusammenwirken wie folgt charakterisieren: Im Kontext der digitalisierungsbezogenen Professionalisierung von Schulleitungen durch die LiS ist eine (gemeinsame) Handlungskoordination nur für die LiS obligatorisch und im (ministeriellen) Auftrag inbegriffen. Für Schulleitungen ist dieses Zusammenwirken fakultativ bzw. in eigenem Ermessen mit Blick auf den je individuellen bzw. einzelschulischen Professionalisierungsbedarf zu gestalten. Somit ist hier auch grundlegend zu fragen, inwiefern bzw. bei welchen Schulleitungen und unter welchen Voraussetzungen Interaktionen mit den LiS im Sinne einer Handlungskoordination überhaupt stattfinden.

### Forschungsstand

#### Forschung und Desiderata zu LiS und zur Fortbildungsadministration

Die wenigen Beiträge, die den Akteur LiS fokussieren, nehmen denselben entweder theoretisch bezüglich seiner Transferaufgabe (vgl. Dehmel 2019) oder anhand „offizieller Selbstdarstellungen und formaler Aufgabenbeschreibungen“ im Sinne eines „Struktur-Überblick[s]“ (Rürup 2014, S. 207) in den Blick, „der die kommunikativ-interaktive Vielfalt der Handlungspraxis von Landesinstituten und ihren Mitarbeiterinnen und Mitarbeitern prinzipiell ausblendet“ (Rürup 2014, S. 207). So werden auch hier entsprechende Forschungslücken markiert: „Wie die einzelnen Landesinstitute […] mit diesen Herausforderungen umgehen und welche Transferstrategien und -maßnahmen sie […] entwickeln werden, bleibt abzuwarten“ (Dehmel 2019, S. 18). Kongruent konstatiert auch Rürup (2014): Die LiS „agieren im Wesentlichen fremdbestimmt als eng an das Schulministerium angebundene Dienstleister. Inwieweit hierbei das somit nahegelegte hierarchische Weisungsverhältnis auch faktisch den Arbeitsalltag bestimmt, ist sicherlich offen“ (Rürup 2014, S. 213).

Zum Fortbildungssystem insgesamt finden sich v. a. Studien zu einzelnen Bundesländern, die auf suboptimale Systemstrukturen hindeuten. Erkenntnisse aus Experteninterviews mit Verantwortlichen des Baden-Württembergischen LiS^2^ deuten darauf hin, dass die Akteure ihre Aufgaben als Umsetzung eines „bildungspolitischen Rahmen[s]“ (Cramer et al. 2019, S. 29) verstehen und im Vergleich dazu eine „nachfrageorientierte Lehrerfortbildung in ‚deutlich geringerem Umfang‘ […] umgesetzt werde“ (Cramer et al. 2019, S. 30). Die interviewten Schulleitungen beschreiben eine je nach Schulgröße unterschiedlich aufwändige schulinterne Fortbildungsplanung und es wird die Bedeutung der Schulleitung für die Schul- und Personalentwicklung und die dazu nötigen Ressourcen betont (Cramer et al. 2019, S. 32). In der „Evaluation zur Lehrerfortbildung in NRW“ urteilen lediglich etwa 25 % der Befragten, „dass die individuellen Bedarfe bzw. die Bedarfe der Schule vollständig abgedeckt“ seien (Altrichter et al. 2019, S. 10). Darüber hinaus wird kritisiert, dass Fortbildungsangebote sehr lange entwickelt werden und die Steuerung personeller Ressourcen hierbei nicht immer angemessen sei (Altrichter et al. 2019, S. 7).

Übergreifend zeichnet sich in beiden Studien – neben einer grundsätzlichen Ressourcenproblematik im Kontext der Professionalisierungsaufgabe bei Schulleitungen – ein Spannungsfeld zwischen zentraler Steuerung und lokal- resp. schulspezifischen Bedarfen ab. Diesbezüglich gilt es „offenbar auch die Frage danach zu klären, wie genau die Bildungsadministration mit ihrer Steuerungshoheit mit Blick auf Lehrerfortbildungen den sich […] ergebenden Fortbildungsbedarf in die Planung mit aufnehmen und mit den berechtigten Anliegen einer zentralen Steuerung verbinden kann“ (Cramer et al. 2019, S. 72). Bezogen auf dieses Spannungsfeld werden entsprechende Desiderata formuliert: Es „bleibt offen, wie sich die zentralen Bedarfe, die auf der einen Seite von der Bildungsadministration identifiziert und gesetzt werden, zum realen individuellen Bedarf einzelner Lehrpersonen oder Schulen auf der anderen Seite verhalten“ (Cramer et al. 2019, S. 72). Insgesamt „kann daher von einer ‚Schnittstellenproblematik‘ ausgegangen werden, deren Bearbeitung ein Desiderat darstellt“ (Cramer et al. 2019, S. 71).

#### Ausgewählte Befunde zur Schulleitung

Die folgenden Forschungsbefunde dienen zum einen der empirischen Charakterisierung genereller Handlungslogiken der Schulleitungsposition und deren Bedingungen. Zum anderen werden spezifische, digitalisierungsbezogene Befunde zum Akteur präsentiert. Beide Facetten des Forschungsstands werden in der Diskussion der Ergebnisse dieser Studie aufgegriffen (Kap. 5), auch um der Monoperspektivität dieser Studie zu begegnen (vgl. Kap. 3.3).

Die o. g. zentrale Position der Schulleitung lässt sich auch empirisch fundieren: Ihr Handeln beeinflusst maßgeblich diverse Facetten der Organisation Schule, steuert Prozesse der Qualitätsentwicklung und wirkt sich indirekt auf den Lernerfolg von Schüler:innen aus (u. a. Pietsch und Tulowitzki 2017; Leithwood und Louis 2012). Die Handlungsfelder von Schulleitenden in Deutschland umfassen die folgenden Bereiche und damit vielfältige Verantwortungsgebiete (vgl. Abb. 1):

In Deutschland beanspruchen administrative Belange und der eigene Unterricht einen erheblichen Teil ihrer Arbeitszeit (48 %), während unterrichtsbezogene Führungstätigkeiten mit 20 % einen geringeren Anteil einnehmen und Personal- und Organisationsentwicklung zusammen auf ca. 16 % kommen (Brauckmann 2014, S. 35). Schulleitungen sehen sich insgesamt einer hohen Belastung ausgesetzt, was sich während der Corona-Pandemie verstärkt zeigt. So geben 70 % der bei Dadaczynski et al. (2021) befragten Schulleitungen an, „in einem für sie belastendem Arbeitstempo zu arbeiten, welches sie nicht dauerhaft durchhalten könnten“ (S. III). Im Gegensatz zu den als bewältigbar eingeschätzten administrativen Tätigkeiten scheinen zur Organisationsführung und -entwicklung, zur Umsetzung von Steuerungsinstrumenten (z. B. Vor- und Nachbereitung von Schulinspektionen) und zur unterrichtsbezogenen Führungsarbeit höhere Unterstützungsbedarfe zu bestehen (Schwanenberg et al. 2018). Vor diesem Hintergrund gibt es Hinweise auf eine Handlungslogik im Sinne einer „Priorisierung der Sicherstellung des Schulalltags […], wodurch schulqualitätsrelevante Aufgaben systematisch vernachlässigt werden“ (Kruse und Huber 2021, S. 394). Ein Blick auf die Berufswahlmotive zeigt indes, dass das Erproben und Entwickeln neuer Ideen als besonders relevant für die Ergreifung des Amts bewertet werden (Cramer et al. 2021, S. 140).

Um Transformationsprozesse in ihrer Funktion als „‚Gatekeeper‘ schulischer Innovationen“ (Klein und Tulowitzki 2020, S. 260) zu initiieren und professionell begleiten zu können, werden Schulleitenden umfangreiche Kompetenzen abverlangt. So erscheint nachvollziehbar, dass sie Fortbildungen in den Bereichen Qualitätsentwicklung und Personalführung tendenziell häufiger besuchen (Schwanenberg et al. 2018). Auch schätzen Schulleitungen die Schwierigkeiten in allen übergeordneten Bereichen in Bezug auf Digitalisierung (infrastrukturelle, pädagogische/didaktische Herausforderungen und bzgl. Kompetenzen und Einstellungen) als sehr hoch oder hoch ein (Schwanenberg et al. 2018). Aktuellere Studien unterstreichen dieses Bild. So deuten entsprechende Ergebnisse darauf hin, dass Schulleitende vor dem Hintergrund der COVID-19 Pandemie zwar positive Entwicklungen der digitalen Ausstattung und Kompetenzen verzeichnen, die Herausforderungen und Belastungen in Bezug auf die Digitalisierung aber dennoch als hoch einschätzen (Feldhoff et al. 2022).

## Fragestellung und Forschungsdesign

### Fragestellung

Angesichts der zentralen Bedeutung der Schulleitung und dem im Forschungsstand konstatierten Unterstützungs- und Qualifizierungsbedarf hinsichtlich digitalisierungsbezogener Schulentwicklung nehmen wir die für diesen Bedarf vornehmlich zuständige Organisation der LiS theoriebezogen (Kap. 2.1) sowohl hinsichtlich ihrer Qualifizierungs- als auch Steuerungsfunktion in den Blick. Gleichzeitig besteht ein Desiderat zur Handlungskoordination und zu zugrundeliegenden Handlungslogiken der LiS (Kap. 2.2.1), besonders bzgl. dieser potenziell gegenläufigen Funktionen. Interpretiert man nun das Agieren der LiS auch als Steuerungshandeln, das mittels der Kompetenzentwicklung von Schulleitungen auf eine gelingende Transformation der Organisation Schule abzielt, gerät die beschriebene Schnittstellenproblematik im Mehrebenensystem (Kap. 2.1 und 2.2.1) in den Fokus. Diesbezüglich und für die Lehrkräftefortbildung allgemein postuliert Altrichter (2019) als Herausforderung u. a. Akzeptanzprobleme bzw. Spannungen bei Fortbildungen zwischen Kompetenzzuwächsen und der „Vermittlung normativer Orientierungen“ (S. 73).

Inwiefern dieses oder andere Phänomene sich in den Handlungslogiken realiter und am Beispiel der digitalisierungsbezogenen Professionalisierung von Schulleitungen zeigen, gilt es nun ausgehend von der theoretischen Argumentation empirisch zu prüfen. Fokussiert man die Schnittstelle zur Schulleitung mit der Beforschung der Perspektive der LiS, so ergeben sich vor dem Hintergrund der Educational Governance folgende Forschungsfragen: Wie lässt sich die Handlungslogik der LiS bezogen auf digitalisierungsbezogene Professionalisierungsangebote für Schulleitungen beschreiben (und ggf. erklären)? Wie gestaltet sich die Handlungskoordination an der ebenenübergreifenden Schnittstelle zur Schulleitung im Sinne von Passungen, Herausforderungen und Professionalisierungsbedarfen?

### Stichprobe, Erhebungs- und Auswertungsmethode

Die zugrunde liegenden Daten sind Interviewtranskripte aus einem Forschungsprojekt zu Impulsen zu digitalisierungsbezogener Schulentwicklung (Tulowitzki et al. 2021), die einer Sekundäranalyse unterzogen wurden. Im Rahmen halbstrukturierter Leitfadeninterviews wurden im Sommer und Herbst 2021 sieben führende Vertreter:innen von LiS (auf vergleichbarer Hierarchieebene) aus je verschiedenen Bundesländern zu digitalisierungsbezogenen Professionalisierungsstrukturen und -angeboten für Schulleitende befragt. Neben der Konzeption, Gestaltung und Passung spezifischer Maßnahmen wurden in den Interviews auch Fragestellungen im Kontext digitaler Schulentwicklungsprozesse, der Koordination, Verantwortungsbereiche verschiedener Akteure und deren Herausforderungen thematisiert. Der Interviewleitfaden wurde situationsbezogen auf den jeweiligen Kontext der Befragten angepasst, um ein flexibles Eingehen auf thematische Schwerpunktsetzungen der Akteure zu ermöglichen. Für die vorliegende Studie wurden die Interviewdaten in einem iterativen Verfahren neu kodiert (vollständige Kodierung durch eine Person, partielle Gegenkodierung durch eine weitere Person mit anschließendem Vergleich und Revision) und mittels qualitativer Inhaltsanalyse ausgewertet (Mayring 2015). Die zentralen Ebenen (LiS, Schule, vgl. Kap. 2.1) wurden dabei als Hauptkategorien definiert und die Auswertung entlang einer typisierenden Strukturierung mit deduktiver Kategorienanwendung auf der ersten Kordierebene vorgenommen (Mayring 2015). Diese Hauptkategorien erscheinen zur Analyse zielführend, weil neben der (theoriebezogenen) Passung (Mehrebenensystem) dadurch eine analytische Trennung zwischen Selbstbeschreibungen (Ebene LiS) und Fremdbeschreibungen (Ebene Schule) ermöglicht wird. Die Darstellung der Ergebnisse der vorliegenden Sekundäranalyse orientiert sich an den beiden Hauptkategorien (vgl. Tab. 1). Durch dieses Vorgehen können in der sich anschließenden Diskussion aus den Selbst- und Fremdbeschreibungen, aus den darin eingelagerten Rollenzuschreibungen und Erwartungshaltungen seitens der LiS, Hinweise zur eigenen sowie beobachteten/antizipierten Handlungslogik abgeleitet werden. Gleichwohl wird in der Diskussion kritisch berücksichtigt, dass auch die Fremdbeschreibungen zur Schule resp. Schulleitung Einblicke in die Handlungslogik der LiS selbst bieten (vgl. Kap. 5). Der Fokus auf *führende* Vertreter:innen der LiS als Expert:innen verspricht hierzu Erkenntnisse, weil sie als Führungskräfte über besonders umfangreiches organisationales Wissen verfügen.

**Table 1:** 

Hauptkategorie	Definition	Ankerbeispiel
LiS (intermediäre Ebene)	Selbstbeschreibungen der LiS zu Gestaltung, Ausbringung und Passungsherstellung von digitalisierungsbezogenen Professionalisierungsangeboten für Schulleitende	*„Also wir sind ja behördlich verortet und unser Auftraggeber ist zunächst einmal das […] Kultusministerium. Das heißt, wir haben natürlich auch ein Steuerungsinteresse des Landes und wir entwickeln Produkte, also rein strukturell gedacht, wir entwickeln eben Produkte, wenn wir eben Aufträge bekommen“ (Int5).*
Schule (Mesoebene)	Einschätzungen der LiS zu Bedarfen und Herausforderungen bzgl. digitalisierungsbezogener Professionalisierungsangebote auf Ebene der Schule	*„Ich glaube […], dass es da gut funktioniert, wo es dann ein schlüssiges Miteinander wird. Wo es eben wirklich arbeitsteilig, wo die Lehrkräfte sich mitgenommen fühlen […], wie wichtig auch die Rollen von Schülerinnen und Schülern, von Eltern sind, also wenn eine Gesamtstimmung in einer Schule ist, dass man sich auf den Weg macht, dass man Dinge verändern will“ (Int2).*

### Grenzen der Studie

Die Studie ist verschiedenfach limitiert: Wenngleich die ursprüngliche Erhebung Aspekte der Zusammenarbeit und Koordination verschiedener Ebenen berücksichtigte, so war diese nicht explizit als Forschung aus einer Governance-Perspektive heraus angelegt. Ferner ist die Aussagekraft der Ergebnisse durch die Stichprobe dahingehend begrenzt, dass nicht alle Bundesländer darin vertreten sind und damit die Handlungsvielfalt des Feldes ggf. nicht vollständig in den Blick gerät. Außerdem bleibt einschränkend zu berücksichtigen, dass es sich bei den Aussagen um Einschätzungen der führenden Vertreter:innen von LiS handelt, die in unterschiedlichen Formen an der digitalisierungsbezogenen Professionalisierung von Schulleitenden beteiligt sind. Zum einen bleibt damit partiell ungeklärt, ob und inwiefern deren Vorstellungen zur eigenen Handlungslogik von anderen planenden, organisierenden Mitarbeitenden und ggf. auch operativen Fortbildungspersonen abweicht. Zudem kann die Frage nach der Handlungskoordination an der Schnittstelle (vgl. Kap. 3.1) hier nur aus der Perspektive der LiS bearbeitet werden. So können wir in diesem Beitrag lediglich die (intendierte) Passungsherstellung von Professionalisierungsangeboten in der Hauptkategorie LiS und Bedarfe und Herausforderungen auf Schulleitungsseite *in der Fremdwahrnehmung durch die LiS* in der zweiten Hauptkategorie in den Blick nehmen. Beides wäre in weitergehenden Studien um die Erforschung der Perspektiven von Schulleitenden und Lehrkräften zu komplementieren.

## Ergebnisse

### Ebene Landesinstitute

Wie in Kap. 2.1 konzeptionell beschrieben und im Ankerbeispiel zu dieser Hauptkategorie (Tab. 1) verbal bestätigt, handeln die LiS grundsätzlich im Auftrag des jeweils übergeordneten Ministeriums. Gleichzeitig wird deutlich, dass ihr Auftrag auch darin besteht, die Passung von Professionalisierungsmaßnahmen an die Bedarfe und Möglichkeiten der schulischen Praxis sicherzustellen, wie die folgende Aussage exemplarisch illustriert:

Wir hören natürlich auch von Schulleitungen oder von zu Qualifizierenden, von Interessierten verschiedene Bedarfe. Auch aus den Evaluationen von Qualifizierungen entnehmen wir Bedarfe und aufgrund dieser Bedarfe treten wir eben in den Austausch und versuchen eben die Aufträge [des Ministeriums, d. A.] weiterzuentwickeln (Int5).

Insofern werden die Projekte auch durch die kontinuierliche Rückmeldung der Teilnehmenden weiterentwickelt und so an den Bedarfen der Schulen ausgerichtet. Die befragten Vertreter:innen der LiS stellen den Austausch mit der Zielgruppe der Professionalisierungsmaßnahmen als Indikator für Anpassungsbedarfe in der Ausrichtung spezifischer Maßnahmen als dienliches Mittel dar. In diesem Zusammenhang wird konkretisiert:

Fortbildner reagieren immer dann, wenn ich zu einem bestimmten Thema nicht nur von einer Schule eine Meldung kriege, das kann man vielleicht ja noch als Einzelerscheinung wahrnehmen, sondern wenn ich in einem größeren Ausmaß, möglicherweise von verschiedenen Schulformen das Signal kriege, hör mal da müssen wir was tun (Int6).

Hierbei scheinen zum einen informelle Kommunikationsweisen aber auch umfangreiche Befragungen und Evaluationen relevant zu sein. Oft wird eine bedarfsorientierte Vorgehensweise in der Initiation von Professionalisierungsmaßnahmen als bedeutsam eingeschätzt, um den sich schnell wandelnden Ansprüchen schulischer Digitalisierungsprozesse gerecht zu werden. Dass dies mit Abstimmungsprozessen innerhalb der LiS verbunden ist, verdeutlicht folgende Aussage:

Die Schulen rufen an, fragen, höre mal ich brauche […] das und das hast du das? Wo ich dann sage, habe ich nicht und dann habe ich meistens Ideen, wie man das dann aufbauen kann und dann muss ich zu den [Kollegen, d. A.] und sage, sag mal passt das für euch in den und den Kurstag oder kriege ich einen, oder wollt ihr das? Und meistens findet das dann einen guten Weg (Int6).

Hierbei wird auch deutlich, dass die LiS nicht nur auf die Artikulation sich dynamisch verändernder Bedarfe der Schulleitenden, sondern insbesondere auch auf deren aktive Fortbildungsbereitschaft maßgeblich angewiesen sind, um dieser mit entsprechenden Angeboten zu begegnen:

Deswegen ist für uns ganz wichtig dass der Impuls vom Schulleiter kommt und wir auch in der Schulleitungsfortbildung so viel zur Verfügung stellen, dass ihm klar ist, dass er der Initiator ist und letztendlich von seiner Ausstrahlung, dass es jetzt für die ganze Schule einen nächsten großen Schritt gibt, der Entwicklung, es auch viel abhängt und wir erst dann wir hilfreich zur Seite stehen können und bei den Umsetzungen ihm Erfahrung aus anderen Schulen vermitteln können, Kontakte vermitteln, Tools anbieten, jede Menge Erfahrung zur Verfügung stellen, aber wenn er nicht ‚Piep‘ sagt, dann kommen wir nicht, weil es muss schon eine bewusste Entscheidung der Schule sein (Int6).

Neben der inhaltlichen Ausrichtung von Professionalisierungsmaßnahmen stellen die Befragten auch die Passung von Form und Umfang der Angebote an die Ressourcen der Teilnehmenden als bedeutsam dar:

Schulleitungen haben so viel zu tun, die sollen einfach sich drei Stunden abknapsen können aber, wenn wir jetzt ein Fortbildungsangebot machen und sagen, so ihr seid elf Tage weg im nächsten Schuljahr, ja dann, ‚hallo, wie soll ich das denn machen‘. Deswegen bieten wir es online an, wegen des Zeitfaktors (Int5).

Zum Teil werden auch Bedarfsmeldungen der Schulleitenden in Bezug zu (geringerem) zeitlichem Umfang oder Online- statt Präsenzveranstaltungen berücksichtigt, die von den Befragten allerdings als weniger wirksam eingeschätzt werden. Auch berichten sie davon, Zugänge zu Informationen sowie Ausschreibungen für Professionalisierungsangebote vor dem Hintergrund begrenzter Kapazitäten der potenziell Teilnehmenden anpassen zu wollen.

Angesichts beschränkter Ressourcen der LiS bestehe ein erhebliches Potenzial darin, Angebote unter Einbezug weiterer Akteure (Universitäten, Stiftungen, etc.) auszurichten. Ebenso wird es auch als sinnvoll angesehen, dass Schulleitungen, als unmittelbar selbst an Innovationsprozessen Beteiligte, Professionalisierungsmaßnahmen moderieren. Gleichzeitig wird auch ein Vernetzungsbedarf zwischen den Schulleitungen wahrgenommen.

### Ebene Schule

Maßnahmen zur Professionalisierung von Schulleitenden zielen nach Aussagen der Befragten nicht nur auf den Kompetenzzuwachs seitens der Schulleitenden selbst, sondern auch auf die Entwicklung der Organisation Schule ab. Schulleitende nehmen in der Einschätzung der Befragten eine herausforderungsreiche Change-Managerfunktion ein, an die entsprechende Erwartungen gestellt werden und die es durch Professionalisierungsmaßnahmen zu stärken gilt.

Dann erhoffen wir uns dadurch auch von der Schule als lernende Organisation insgesamt ein Entwicklungspotenzial, was gerne ausgeschöpft wird und zwar im gemeinsamen Verständnis aller, […] und deswegen setzen wir an, bei Leitungskräften (Int5).

Die Rolle und Funktion der Schulleitung wird unter anderem durch die Interaktion und in Aushandlungsprozessen mit den Akteuren der Schulgemeinschaft hergestellt und definiert. Hier liege eine wichtige Aufgabe der Schulleitung im konstruktiven Umgang mit Widerständen im Kollegium bei der Initiierung von digitalisierungsbezogenen Schulentwicklungsprozessen, deren Umfang schwer abzuschätzen ist. Insofern nehmen die Befragten auch die Schulgemeinschaft und ihre Bereitschaft und Einstellung im Kontext digitaler Schulentwicklungsprozesse als relevanten Faktor im Kontext der langfristigen Wirksamkeit von Professionalisierungsmaßnahmen in den Blick (siehe Ankerzitat, Hauptkategorie Schule, Tab. 1).

Gleichzeitig wird bereits in diesen ersten Zitaten deutlich, dass die Aussagen zu dieser Hauptkategorie – definitionsgemäß zu „Bedarfen und Herausforderungen […] der Schule“ (Tab. 1) – oftmals durchsetzt sind von normativen Erwartungen aus einer Fortbildungsperspektive an das Handeln verschiedener Akteure auf der Schulebene. Weil sich diese Interpretationslinie in allen Aussagen zur Hauptkategorie „Ebene Schule“ finden lässt, wird darauf in der Diskussion (Kap. 5) übergreifend eingegangen.

Im Kontext digitaler Schulentwicklungsprozesse sehen die Befragten die Schulleitungen in der Pflicht, sich mit technischen Möglichkeiten und dem Einsatz im Unterricht und der Organisation auseinanderzusetzen, um Prozesse kompetent initiieren zu können, aber auch, um gemeinsame, innovationsoffene Wertorientierungen an der Schule zu fördern. Jene Wertorientierungen, oft mit „Mindset“ oder „Haltung“ beschrieben, stellen aus der Perspektive der Befragten eine zentrale Gelingensbedingung für (digitalisierungsbezogene) Schulentwicklungsprozesse dar. Diese Orientierungen werden daher auch Gegenstand von Professionalisierungsmaßnahmen.

Also die Herausforderungen, die beziehen sich auf alle Bereiche, auf alle Bereiche, auf alle Beteiligte, auf alle Prozesse und vor allen Dingen auch auf die Haltung, auf das Mindset, das notwendig ist […] Handlungsfelder von Schulleitung. Die Veränderungen betreffen alle Handlungsfelder, und zwar komplett (Int5).

Im Zuge dessen wird die Professionalisierungsarbeit an einer solchen Grundhaltung gegenüber digitalen Transformationsprozessen als große Herausforderung beschrieben, um langfristige Wirkung und Veränderungsprozesse innerhalb der Schulen zu ermöglichen:

Es ist wirklich eine Transformation. Es ist für mich noch einmal einen Schritt weiter. Das ergreift alles in der Schule […]. Das muss man einfach so sehen […] diese Gesamtkomplexität, die können ganz viele Schulleitungen nicht sehen und wissen nicht, wo sie anfangen sollen und da sehe ich halt ein großes Problem, dann geht es immer nur auf diese technische Schiene (Int3).

Als relevant wird folglich die Verknüpfung von digitalisierungsbezogenen Herausforderungen mit weiteren Querschnittsaufgaben im Sinne umfassender Schulentwicklungsprozesse als auch das Verständnis von Digitalisierung als konstanter und zukunftsrelevanter Aspekt des schulischen Alltags angesehen. Nach Aussagen der Befragten ergeben sich Herausforderungen in der Motivation und Überzeugung der Schulleitenden, digitale Veränderungsprozesse der eigenen Schulen im Sinne eines umgreifenden Verständnisses zu initiieren.

Da glaube ich ist ein großer Bedarf, […] dass es eben kein entweder oder ist. Dass gerade Auseinandersetzung mit den Möglichkeiten der Digitalisierung eben auch Chancen sind, da eben Dinge zu vereinfachen und zusammen zu führen und Ressourcen, […] frei zu legen, weil eben Digitalisierung unterstützen kann, aber da ist eine große Hemmnis, da wird es immer wieder als ein ‚wie soll ich das jetzt auch noch schaffen‘ gesehen (Int2).

Ansätze, um entsprechende Aushandlungsprozesse zu begleiten, sehen die Befragten darin, neben Schulleitenden auch weitere Mitglieder des Leitungsteams oder Kollegiums gemeinsam zu schulen.

Kernidee war, dieses Team bringt es als Team zurück an die Schule, deswegen nicht an einzelner Digitalbeauftragter oder Informatik- oder Medienlehrkraft, die dann als Einzelkämpfer nicht in die Breite wirkt, sondern genau diese Mischung aus der Fachkompetenz und der Schulleitung (Int2).

Hierbei werden Schulformspezifika als bedeutsam für die digitalisierungsbezogenen Herausforderungen der Schule (und damit auch für die Passung von Professionalisierungsmaßnahmen) wahrgenommen. So sei es ein entscheidender Unterschied für die Fortbildung, inwiefern die Schulleitung an ihrer Schule auf ein Team mit dezidierter Funktion zur (digitalisierungsbezogenen) Schulentwicklung zurückgreifen kann, was an kleineren Schulen, oftmals Grundschulen, häufig nicht gegeben sei.

## Diskussion

Zur Handlungslogik der LiS bzgl. digitalisierungsbezogener Professionalisierungsmaßnahmen für Schulleitungen zeigt sich, dass diese gerahmt wird von Steuerungsimpulsen der Landesebene und von den Bedarfen auf Schulebene, auch bezüglich der Schulform und -größe (siehe auch Cramer et al. 2019, Kap. 2.2.1). Die konkreten Angebote unterliegen insofern in der Ausrichtung und Gestaltung grundsätzlich den Vorgaben der Länder, dies sind jedoch mitnichten die einzigen „Relevanzkriterien und Informationsanforderungen, innerhalb derer die Akteure […] Informationen und Wissen generieren, ausdeuten, gewichten und verteilen“ (Definition Handlungslogik Kap. 2.1, Kussau und Brüsemeister 2007, S. 33). Vielmehr verdeutlichen die Ergebnisse, dass neben den Ressourcen der LiS auch die antizipierten oder (z. T. systematisch) eingeholten Bedarfe und Möglichkeiten der Schulleitungen zu Professionalisierungsmaßnahmen eine zentrale Bedeutung in deren Ausgestaltung durch die LiS erfahren (Kap. 4.1). Das erscheint plausibel, da – wie auch im Interviewzitat im Untertitel dieses Beitrags deutlich wird – Schulleitungen in der Regel weder institutionell verpflichtet noch in sonstiger Weise zu einer Weiterqualifikation (per Zwang oder Verhandlung) „gesteuert“ werden können. Insofern sind für die Herstellung von Passung an der Schnittstelle im Sinne einer gelingenden Handlungskoordination zwischen LiS und Schulleitung Professionalisierungsangebote nötig, die sowohl inhaltlich als auch hinsichtlich des Formats für die Handlungslogik der Schulleitung anschlussfähig sind. Deshalb priorisieren die LiS in ihrer Handlungslogik die Beobachtung des Akteurs der Schulleitung, um auf dieser Basis passende Angebote zu konzipieren.

Gleichzeitig zeigen sich in den Ergebnissen Hinweise auf die von Altrichter (2019) postulierte Spannung zwischen Kompetenzzuwächsen und der „Vermittlung normativer Orientierungen“ (S. 73, vgl. Kap. 3), denn neben den ministeriellen Vorgaben, eigenen Ressourcen und der Passung zur Handlungslogik der Schulleitung sind auch eigene Relevanzsetzungen im Sinne dieser normativen Orientierungen handlungsleitend für die LiS. Als maßgeblich wird von den Interviewten hier eine innovationsförderliche „Haltung“, ein „Mindset“ unter Schulleitungen sowohl als Gelingensbedingung als auch als Gegenstand der Professionalisierungsmaßnahmen benannt. Schließt man sich der Forderung der hier interviewten Akteure der LiS bezüglich einer innovationsförderlichen normativen Orientierung der Schulleitungen an, ist darin aus governancetheoretischer Perspektive ein Steuerungsproblem zu identifizieren: Denn dann entziehen sich diejenigen Schulleitungen, die aufgrund einer abweichenden normativen Orientierung (hier: unzureichende Innovationsbereitschaft) nicht an Professionalisierungsangeboten zur digitalisierungsbezogenen Schulentwicklung teilnehmen, der Steuerungsmöglichkeit durch Fortbildungsangebote zur Bearbeitung ebendieser normativen Orientierung. Anders ausgedrückt: Schulleitungen, bei denen keine Bereitschaft zur digitalisierungsbezogenen Schulentwicklung besteht, können auch kaum durch Professionalisierungsmaßnahmen der LiS dazu „gesteuert“ werden (vgl. zur steuerungsskeptischen Perspektive Kap. 2.1). Vor dem Hintergrund des Forschungsstands zum Akteur der Schulleitung sollten jedoch auch andere Erklärungsansätze herangezogen werden: Zahlreiche Aufgaben in den Handlungsfeldern der Schulleitung, Belastungen, Herausforderungen und eine Logik der Priorisierung der Sicherstellung des Schulalltags (Kap. 2.2.2) kommen als Argumente für eine fehlende Passung der Angebote an der Schnittstelle ebenfalls in Betracht und werden von den LiS auch ähnlich wahrgenommen (Kap. 4.1). Diese Argumentation ließe sich zwar auch als Rechtfertigung der LiS gegen externe Kritik zur Passung der Angebote interpretieren, gleichzeitig liegen Befunde zur Ressourcen-, insbesondere Zeitknappheit der Schulleitung (vgl. Kap. 2.2.2) vor, die eine solche Auslegung als tendenziell weniger angemessen erscheinen lassen. Und auch bei Cramer et al. (2019, s. o.) wird die Bedeutung hinreichender Ressourcen auf der Schulebene hervorgehoben. Der Versuch der Anpassung des Formats der Professionalisierungsangebote an diese als sehr begrenzt wahrgenommenen Ressourcen der Schulleitung lässt sich in dem Zusammenhang als Koordinierungsstrategie anführen. Allerdings sind darin ebenfalls transintentionale Effekte zu vermuten: Kurze, zusammenhangslose Fortbildungselemente ohne die Balance von Input‑, Anwendungs- und Reflexionsphasen sind vor dem Hintergrund der Fortbildungsforschung als eingeschränkt wirksam einzuschätzen (Lipowsky und Rzejak 2021), was sich analog in der Skepsis der LiS bezüglich kurzer Formate zeigt (vgl. Kap. 4.1). Dies gilt insbesondere dann, wenn im Rahmen von Professionalisierungsangeboten nicht nur separat schulbare technische Kompetenzen, sondern umfassende (die Komplexität der Handlungsfelder insgesamt betreffende) Handlungsstrategien oder normative Orientierungen zum Professionalisierungsgegenstand werden sollen, wie es die Ergebnisse zur Schulebene (Kap. 4.2) nahelegen.

Zu den Ergebnissen der zweiten Hauptkategorie (Kap. 4.2) ist diskussionswürdig, dass insbesondere an der Schnittstelle verortete Passungen, Bedarfe und Herausforderungen in der Beschreibung seitens der LiS durch eine normative Fortbildungsperspektive gefärbt erscheinen. Zum einen ist damit die benannte Limitation der Monoperspektivität dieser Studie angesprochen (Kap. 3.3). Zum anderen deutet diese Interpretation darauf hin, dass die oben diskutierten normativen Orientierungen nicht nur *Gegenstand* der Professionalisierungsangebote und in der beschriebenen Weise schulleitungsseitig *Voraussetzung* zur Nutzung dieser Angebote sind, sondern gleichzeitig durchgängig als *Erwartung* seitens der LiS die Schnittstelleninteraktionen prägen können. Die in Kap. 4.2 angeführten Aspekte wären in dieser Lesart als Rollenerwartungen an die Schulleitung zu verstehen, zu denen sich die schulischen Akteure implizit oder explizit positionieren. Versteht sich eine Schule (wohlgemerkt in der Wahrnehmung durch die LiS) beispielsweise nicht als „lernende Organisation […] im gemeinsamen Verständnis aller“ (Int5) oder sieht eine Schulleitung nicht die „Gesamtkomplexität“ (Int3) der digitalisierungsbezogenen Schulentwicklung, so wäre damit zunächst die Erwartung der LiS an die Rolle der Schulleitung resp. an die der Lehrkräfte enttäuscht. Welche Auswirkungen eine solche, potenzielle Enttäuschung auf die Handlungskoordination an der Schnittstelle im Fortbildungssystem hat, wäre in weiteren Studien zu erforschen.

Abstrahiert lassen sich somit die Forschungsfragen nach der Handlungslogik der LiS sowie nach der Handlungskoordination an der ebenenübergreifenden Schnittstelle im Kontext digitalisierungsbezogener Professionalisierungsmaßnahmen für Schulleitungen wie folgt beantworten: Die LiS nehmen Bedarfe der Schulleitungen (im Modus der Beobachtung) wahr und passen die Professionalisierungsangebote (unter Berücksichtigung weiterer Aspekte, u. a. der Steuerungsimpulse der Landesministerien) entsprechend an. Die Schulleitung ist damit in der Position, durch die Kommunikation eigener Bedarfe die Angebote beeinflussen zu können und zudem – im Rahmen ihrer eigenen Handlungslogik und Priorisierung – entsprechende Angebote (nicht) auszuwählen. Die Steuerung von Handlungskoordination im Sinne der „Art und Funktionalität des Zusammenwirkens“ (Kap. 2.1, Altrichter und Maag Merki 2016, S. 9) in dieser Schnittstelle kann somit in erheblichem Maße beim Akteur der Schulleitung verortet werden. Wenngleich die LiS in ihrer in dieser Studie auch empirisch belegbaren Qualifizierungs‑, Unterstützungs- sowie Steuerungsfunktion über einen bedeutsamen Gestaltungsspielraum verfügen, so können diese Funktionen erst auf Aktivität der Schulleitung hin ihre potenzielle Wirksamkeit entfalten, wie das zuvor erwähnte Interviewzitat abschließend verdeutlicht:

Aber wenn er nicht ‚Piep‘ sagt, dann kommen wir nicht, weil es muss schon eine bewusste Entscheidung der Schule sein (Int6).

Somit zeigt sich in dieser Studie empirisch und differenziert am Beispiel der digitalisierungsbezogenen Schulleitungsprofessionalisierung, dass es zur Ausschöpfung der „Unterstützungs- und Steuerungsfunktion“ des Fortbildungssystems „einer Abstimmung der Aufgaben und Rollen dieses ‚intermediären Akteurs‘ mit jenen anderer Systemakteure“ (Altrichter et al. 2019, S. 7) bedarf. In dieser Abstimmung besteht folglich eine wichtige Aufgabe und Herausforderung für die LiS zur (digitalisierungsbezogenen) Professionalisierung von Schulleitungen wie auch von Lehrkräften insgesamt.

## Fazit und Implikationen

In dieser Studie wurden zentrale Desiderata aus der Forschung zum Akteur der LiS sowie zur Fortbildungsadministration insgesamt bearbeitet. Aus den diskutierten Erkenntnissen leiten wir die folgenden Implikationen ab:

Angesichts der Forschung zur Schulleitungsposition ergibt sich eine hohe Motivation dieses Akteurs bezüglich der gestalterischen Aufgaben in der Führung der Organisation Schule (Kap. 2.2.2). Das lässt uns vermuten, dass die LiS in der Interaktion mit der Schulleitung verstärkte Passung durch die Angebote herstellen könnten, indem sie nicht nur auf das Potenzial zum effizienterem Ressourceneinsatz (vgl. Kap. 4.2) durch digitalisierungsbezogene Schulentwicklung hinweisen, sondern auch die Rolle der Schulleitung als Gestaltende im Sinne eines motivierenden Anreizes in den Ausschreibungen der Angebote stärken. Für die Schulleitungen gilt es umgekehrt, sich ihrer Steuerungsfunktion bezüglich der Angebotsgestaltung zunächst bewusst zu sein und gezielt eigene Unterstützungs- und Professionalisierungsbedarfe zu identifizieren und in Interaktion mit den LiS zu kommunizieren. Hierbei wäre die eigene Innovationsbereitschaft mit Blick auf die Kapazitäten des Kollegiums (der eigenen Organisation) sowie hinsichtlich des Gestaltungsspielraums, auch für eine effizienzsteigernde, ressourcenfreisetzende Umsetzung digitalisierungsbezogener Schulentwicklung kritisch in den Blick zu nehmen. Aber auch auf der Makroebene gilt es, die Regelungsstrukturen im Schulsystem hinsichtlich der Ressourcen, Anreize und Verbindlichkeiten für Schulleitungsfortbildungen zu prüfen. Hier scheint die Schaffung zeitlicher Freiräume für die Professionalisierung von Schulleitungen angesichts der Forschung zur Belastung, Aufgaben und (notwendigen) Priorisierungen von Schulleitungen (Kap. 2.2.2) für die Ermöglichung wirksamer Fortbildungen (Kap. 5) geboten. Die steuerungsskeptisch stimmenden Befunde (Kap. 5) verweisen darüber hinaus auf die Bedeutung der Personal*auswahl*verfahren für die Schulleitungsposition, die sich auf Systemebene hinsichtlich des Auswahlkriteriums innovationsförderlicher normativer Orientierungen prüfen ließen.

Eine zumindest theoretische Option wäre der Aufbau von Druck seitens der LiS auf Schulleitende, Fortbildungen zu besuchen, z. B. durch die Einführung entsprechender Verpflichtungs- und Sanktionierungsmechanismen (z. B. Böttger-Beer und Koch 2008). Allerdings könnten Schulleitungen darauf mit angepassten Vermeidungsstrategien (z. B. Krankmeldung, pro forma Teilnahme) reagieren. Insbesondere erscheint es fraglich, ob so Reflexions- und Änderungsimpulse auf die in den Interviews geäußerte Haltung erzielt werden können.

Gleichzeitig sind die normativen Erwartungen seitens der LiS (selbst)kritisch zu hinterfragen. Diese Normen scheinen mal implizit und mal expliziter die Aussagen der hier Befragten zu prägen. Wenn das analog auch für die Schnittstelleninteraktion zuträfe, scheint empfehlenswert, entsprechende Erwartungen in der Interaktion zu explizieren, möglicherweise auch in den (formalen) Ausschreibungen der Fortbildungsangebote. Es wäre dann zentral, dass nicht eine spezifische normative Orientierung (z. B. hinsichtlich einer Haltung oder dem Blick für die Gesamtkomplexität) vorab erwartet oder vorausgesetzt wird, wenn diese gleichzeitig als Gegenstand Ziel einer Professionalisierungsmaßnahme ist. Das setzt zunächst eine konzeptionelle Klärung voraus, von welchem Akteur welche normativen Orientierungen inwiefern erwartet werden können und sollten und welche wiederum erst in der fortschreitenden Professionalisierung zu lernen/zu erwerben sind. Bleibt eine solche Klärung und Explikation aus, könnte ein Risiko darin bestehen, dass LiS den Erfolg eigener Professionalisierungsangebote und -bemühungen als derart voraussetzungsreich deklarieren, dass die Schule/Schulleitung einseitig für die Erfüllung der Voraussetzung (hier z. B. das Vorhandensein einer gewissen „Haltung“) angesprochen wird. Anders formuliert: Es gilt, durch konzeptionelle Klärungen zu vermeiden, dass vorab von Schulleitungen erwartet wird, was erst durch kontinuierliche Professionalisierung erreicht werden soll bzw. kann.

Schließend ist zu ergänzen, dass die Steuerung digitalisierungsbezogener Schulentwicklungsprozesse *nicht nur* über die Unterstützung und Professionalisierung von Schulleitenden durch die LiS zu betrachten ist. Zum einen existieren auch zahlreiche Unterstützungsangebote von privatwirtschaftlichen Akteuren und Stiftungen. Zum anderen wird die digitalisierungsbezogene Schulentwicklung maßgeblich von Lehrpersonen umgesetzt, für die ebenfalls Vorgaben und Unterstützungs- resp. Professionalisierungsangebote von verschiedenen Akteuren existieren. Ein ganzheitlicher Blick auf schulische Steuerungs‑, Unterstützungs- und Professionalisierungsprozesse im Digitalisierungskontext erscheint daher für weitergehende Forschung geboten. Die in dieser Studie vorgestellten steuerungsbezogenen Analysen der Perspektive der LiS auf die Schulleitungsprofessionalisierung können hierzu einen Beitrag darstellen.

## Footnotes

1 Darüber hinaus haben die LiS übergreifende und weitere Aufgaben, wie bei Rürup (2014) und auf der Übersichtsseite des deutschen Bildungsservers (2022) ersichtlich ist.

2 In der Studie wurde u. a. die „Landesakademie für Fortbildung und Personalentwicklung an Schulen“ beforscht. Nach dessen Auflösung 2019 ist nun das neugegründete „Zentrum für Schulqualität und Lehrerbildung“ mit den entspr. Aufgaben betraut (vgl. DBS 2022).
